# Electroacupuncture attenuates cognition impairment via anti-neuroinflammation in an Alzheimer’s disease animal model

**DOI:** 10.1186/s12974-019-1665-3

**Published:** 2019-12-13

**Authors:** Mudan Cai, Jun-Hwan Lee, Eun Jin Yang

**Affiliations:** 1 Department of Herbal Medicine Research, Korea Institute of Oriental Medicine, 1672 Yuseong-daero, Yuseong-gu, Daejeon, 305-811 Republic of Korea; 2 Department of Clinical Research, Korea Institute of Oriental Medicine, 1672 Yuseong-daero, Yuseong-gu, Daejeon, 305-811 Republic of Korea

**Keywords:** Alzheimer’s disease, Electroacupuncture, Cognitive function, Neuroinflammation

## Abstract

**Background:**

Alzheimer’s disease (AD) is a neurodegenerative disorder characterized by progressive loss of cognitive abilities and memory leading to dementia. Electroacupuncture (EA) is a complementary alternative medicine approach, applying an electrical current to acupuncture points. In clinical and animal studies, EA causes cognitive improvements in AD and vascular dementia. However, EA-induced changes in cognition and microglia-mediated amyloid β (Aβ) degradation have not been determined yet in AD animals. Therefore, this study investigated the EA-induced molecular mechanisms causing cognitive improvement and anti-inflammatory activity in five familial mutation (5XFAD) mice, an animal model of AD.

**Methods:**

5XFAD mice were bilaterally treated with EA at the Taegye (KI3) acupoints three times per week for 2 weeks. To evaluate the effects of EA treatment on cognitive functions, novel object recognition and Y-maze tests were performed with non-Tg, 5XFAD (Tg), and EA-treated 5XFAD (Tg + KI3) mice. To examine the molecular mechanisms underlying EA effects, western blots, immunohistochemistry, and micro-positron emission tomography scans were performed. Furthermore, we studied synapse ultrastructures with transmission electron microscopy and used electrophysiology to investigate EA effects on synaptic plasticity in 5XFAD mice.

**Results:**

EA treatment significantly improved working memory and synaptic plasticity, alleviated neuroinflammation, and reduced ultrastructural degradation of synapses via upregulation of synaptophysin and postsynaptic density-95 protein in 5XFAD mice. Furthermore, microglia-mediated Aβ deposition was reduced after EA treatment and coincided with a reduction in amyloid precursor protein.

**Conclusions:**

Our findings demonstrate that EA treatment ameliorates cognitive impairment via inhibition of synaptic degeneration and neuroinflammation in a mouse model of AD.

## Background

Alzheimer’s disease (AD) is a neurodegenerative disease characterized by progressive loss of cognitive and memory functions leading to dementia. The main pathological hallmarks of AD are extracellular amyloid plaques, intraneuronal accumulation of hyperphosphorylated tau proteins, and neuroinflammation [1, 2], but the precise pathophysiology in all its complexity remains elusive. Although several attempts have been made to develop drugs that target amyloid plaques or hyperphosphorylated tau proteins, there is still no effective treatment option for AD patients.

Neuroinflammation occurs near amyloid plaques and hyperphosphorylated tau sequesters and leads to micro- and astrogliosis as its pathological markers [3]. Microglial cells are mainly involved in neuroinflammatory processes, and microglia dysfunction is related to AD development [4]. Microglial cells surround amyloid plaques and hyperphosphorylated tau aggregates, and microglial phagocytosis prevents AD progression [5, 6]. Microglia also have critical roles in the regulation of synapse elimination, a critical process for precise synaptic connectivity and synaptic plasticity [7]. In addition, astrocytes activated by AD-associated neuroinflammation also influence synaptic communication [8] and synaptic apoptosis, thus contributing to the initial cognitive decline in AD [9]. Reactive astrocytes produce pro-inflammatory factors including tumor necrosis factor (TNF)-α, interleukin (IL)-1β, and cyclooxygenase-2 (COX2) causing synaptic disturbances and neuritic dystrophy in an AD animal model [10].

Electroacupuncture (EA) is one of the complementary alternative medicine techniques. It uses the acupuncture method by applying an electrical current to acupuncture needles. The effectiveness of EA for cognitive improvement in AD and vascular dementia has been proven in various clinical and animal studies [11, 12]. Some papers reported that the neuroprotective effects induced by EA are related to its anti-inflammatory activity [11, 12]. Feng et al. demonstrated that EA treatment suppresses the expression levels of pro-inflammatory cytokines including IL-1α, IL-6, and TNF-α that are related to the observed cognitive decline [13]. However, the direct influence of EA treatment on cognition and neuroinflammation in AD animals has not been elucidated yet. Therefore, the current study investigated the molecular mechanisms of EA effects causing cognitive improvement and anti-inflammatory actions in an animal model of AD, the transgenic mice expressing five familial mutations (5XFAD) linked to AD. We found that EA treatment attenuates cognitive impairment and neuroinflammation in 5XFAD mice. Furthermore, we demonstrate that EA treatment regulates microglia-mediated Aβ deposition in this animal model of AD. Taken together, we suggest that EA treatment can be useful to improve cognition in AD animals via anti-inflammatory effects.

## Methods

### Animals

5XFAD mice overexpress human amyloid precursor protein (APP) and presenilin-1 (PS1) mutants, namely the Swedish (K670 N, M671 L), Florida (I716V), and London (V717I) mutations in APP and the PS1 mutations M146 L and L286 V. Male 5XFAD mice and female B6/SJL were obtained from the Jackson Laboratory (Bar Harbor, ME, USA) and maintained in the Korea Institute of Oriental Medicine (KIOM). The offspring were genotyped by polymerase chain reaction analysis using tail DNA to identify mice with transgenic genes [14, 15]. Four animals per cage were housed in an environment with a temperature of 21 ± 3 °C and humidity of 50 ± 10% with a 12-h light/dark cycle (light on 07:00–19:00 h). Access to water and food was ad libitum. Female transgenic mice were randomly assigned to two groups, either the group of 5XFAD transgenic mice (Tg) or the group of 5XFAD transgenic mice treated with EA (Tg + KI3). Age-matched littermate B6/SJL mice of the non-transgenic group (non-Tg) were used as control.

All applicable international, national, and/or institutional guidelines for the care and use of animals were followed. Animals were kept in accordance with the animal care guidelines of the KIOM. All experiments were approved by the Institutional Animal Care and Use Committee (IACUC) of KIOM (approval number 18-033).

### Electroacupuncture (EA) treatment

For EA stimulation, 6.5-month-old 5XFAD mice were subjected to EA treatment at the Taegye (KI3) acupoint. The KI3 acupoint was located on the medial aspect of the foot, between the medial malleolus and the Achilles tendon [16]. Acupuncture needles (diameter 0.18 mm, length 8 mm) were purchased from Dongbang Medical (Boryeong, Korea). All mice were anesthetized with 3% isoflurane (Hana Pharm Co. Ltd., Hwaseong, Korea) for induction and maintained during electrical stimulation with 1.5% isoflurane in a mixture of 70% nitrous oxide and 30% oxygen. The anode and cathode of the electrical stimulator (Partner-1; Daejeon, South Korea) were connected to the two acupuncture needles. Acupuncture needles were inserted at a depth of 2 mm, and electrical stimulation pulses were applied for 15 min (1 mA, 2 Hz) at the bilateral KI3 acupoints. EA treatment was performed three times per week for 2 weeks.

### Novel object recognition (NOR) test

The novel object recognition test, including habituation, training, and probe trial, was conducted for 3 days according to previously described methods [17, 18]. On the first day, all mice were exposed individually to the test box (40 × 40 × 40 cm) for 10 min to habituate to the test environment without any objects. On the second day, the animals were placed again in the box to explore two identical familiar objects for 5 min. In the probe test 24 h after the training trial, the animals were placed in the same box, one object was exchanged with a novel object of a different shape but a similar texture, and the mice were given 5 min to examine the items. Boxes were thoroughly cleaned with 70% ethanol to remove residual odors between test runs. The exploration time was recorded using a video camera, and the percentage of exploration time of the novel object was determined according to the following equation: (*T*_novel_)/(*T*_novel_ + *T*_familiar_) × 100. In this behavioral test, the total exploration time (*T*_novel_ + *T*_familiar_) was expected to be > 10 s in the probe trial; if the total exploration time was less than 10 s, this mouse was not included in the analysis.

### Y-maze test

The Y-maze test was performed the day after the last EA treatment as described previously [19]. The Y-maze was a Y-shaped maze made of black polyvinyl chloride plastic (40 cm long and 3 cm wide with 15-cm-high walls) in which the arms are at 120° angles from each other. Mice were put into the center of the maze and allowed to explore it. The numbers of entries and the sequence of entered arms were manually recorded for 8 min. A spontaneous alternation was defined as entries into three different arms consecutively (i.e., ABC, CAB, or BCA, but not BAB and CBC). The Y-maze arms were thoroughly cleaned with 70% ethanol to remove residual odors after each test. The calculation was defined as follows: percentage alternation = (number of alternations)/(total arm entries − 2) × 100. The number of total arm entries served as an indicator of locomotor activity.

### Western blots

Western blotting was performed as described previously [20]. Prefrontal cortexes were lysed in radioimmunoprecipitation assay buffer (Biosesang, Gyeonggi-do, Korea) containing phosphatase and proteinase inhibitors (Thermo Fisher Scientific, Waltham, MA, USA). The total loading protein (20 μg) was quantified using the Bicinchoninic Acid Assay Kit (Pierce, Rockford, IL, USA) and separated on Bolt 4–12% Bis-Tris Plus gels (Thermo Fisher Scientific). The gels were transferred to polyvinylidene difluoride membranes (Bio-Rad, Hercules, CA, USA). Transferred membranes were incubated with primary antibodies overnight at 4 °C. The next day, these membranes were incubated with secondary antibodies (Santa Cruz Biotechnology, Santa Cruz, CA, USA). The immunoblots were visualized using a ChemiDoc Imaging System device (Bio-Rad) and quantified using ImageJ (version 1.46j; National Institutes of Health, Bethesda, MD, USA). The primary antibodies used were as follows: cluster of differentiation 11b (CD11b), heme oxygenase (HO)-1, TNF-α, COX2, and tubulin (all 1:1000; Abcam, Cambridge, MA, USA); B cell lymphoma-2-associated X protein (Bax), transferrin (all 1:1000; Santa Cruz Biotechnology); β-site amyloid precursor protein cleaving enzyme 1 (BACE), postsynaptic density protein 95 (PSD-95; all 1:1000; Cell Signaling Technology, Danvers, MA, USA); APP, apolipoprotein E (APOE; all 1:1000; Merck Millipore, Burlington, MA, USA); and glial fibrillary acidic protein (GFAP, 1:5000; Agilent Technologies, Santa Clara, CA, USA).

### Electrophysiology

Electrophysiology was performed at Dong-A University (Busan, Korea). Mice were anesthetized, and their brains were removed and transferred into cold artificial cerebrospinal fluid (NaCl, 124 mM; KCl, 3 mM; NaHCO_3_, 26 mM; NaH_2_PO_4_, 1.25 mM; CaCl_2_, 2 mM; MgSO_4_, 1 mM; D-glucose, 10 mM; bubbled with 95% O_2_/5% CO_2_) at 24 h after the last EA treatment. The prefrontal cortex regions were isolated and cut into 400-μm thick slices using a vibratome. Field excitatory postsynaptic potentials (fEPSPs) were recorded in layer V of the prefrontal cortex. After observing a stable baseline for 20 min, long-term potentiation (LTP) was induced by high-frequency stimulation (100 pulses at 100 Hz, 5 trains, 10-s intervals between stimulations of the prefrontal cortex), and fEPSPs were recorded at 2-min intervals for 100 min. For quantification, the LTP ratios were averaged for the last 10 min of the fEPSP recordings.

### Aβ_1–42_ enzyme-linked immunosorbent (ELISA) assays

To determine the amounts of insoluble Aβ_1–42_ in the prefrontal cortexes from mice of each group, a beta-amyloid 1–42 quantitative ELISA kit was used (Anaspec Inc., Fremont, CA, USA). The prefrontal cortex tissue was homogenized with guanidine-HCl for 4 h at room temperature, thoroughly mixed, and centrifuged at 12,000 rpm for 15 min. The collected supernatants of the prefrontal cortexes were diluted 1:10000 in sample dilution buffer and measured according to the manufacturer’s protocol.

### Immunohistochemistry and immunofluorescence staining

For immunohistochemistry and immunofluorescence studies, mice were perfused with phosphate-buffered saline (PBS), and their brains were removed. The brains were fixed in 4% paraformaldehyde for 3 days at 4 °C. Fixed brains were embedded in paraffin and sectioned to 10-μm thickness. Immunohistochemistry and immunofluorescence studies were conducted as previously described [21].

For Aβ immunohistochemistry staining, the slices were deparaffinized in xylene, rehydrated in graded alcohol solutions, and washed again in PBS. Sections were incubated in 90% formic acid for 5 min to retrieve the antigens before incubating them with primary antibodies against anti-Aβ 4G8 (1:400; BioLegend, San Diego, CA, USA) overnight at 4 °C. The next day, the sections were incubated with the matched secondary antibody before visualization of the antibody staining with Avidin-Biotin Complex solution (Vector Laboratories, Burlingame, CA, USA) followed by 3,3′-diaminobenzidine peroxidase substrate solution (Vector Laboratories). For quantification of Aβ plaques, images of the slices were captured using a microscope (Olympus BX53, Tokyo, Japan), and the cellSens software was used to analyze the diameter of Aβ plaques in the captured image. In this study, immunoreactive Aβ was counted if an Aβ plaque had a diameter > 40 μm.

For double immunofluorescence staining, brain slices were incubated in formic acid for antigen retrieval and blocked in 5% bovine serum albumin. The tissue samples were incubated overnight at 4 °C with the following primary antibodies: rabbit anti-CD68 antibody (1:500; Abcam) and mouse anti-Aβ (4G8) antibody (1:400; BioLegend). On the next day, the sections were incubated with a secondary antibody conjugated to goat-anti-mouse Alexa 488 or goat-anti-rabbit Alexa 598 (1:1000; Invitrogen, Carlsbad, CA, USA), then washed and mounted on glass slides using Vectashield mounting medium (Vector Laboratories). For quantification, images were taken with a fluorescence microscope (Olympus BX53) and the numbers of Aβ-positive plaques and CD68-positive microglial cells were counted.

### MicroPET scanning

MicroPET imaging was performed at the Osong Medical Innovation Foundation (Osong-eup, Chung Buk, South Korea). An animal positron-emission tomography (PET) scanner (Nano PET/CT; Mediso, Hungary) was used for imaging of mice under inhalation anesthesia (1.5–2.0% of isoflurane in 1.0–1.5 l/min of oxygen). For microPET scanning, mice were food-restricted for over 8 h and anesthetized, and fluorodeoxyglucose (^18^F-FDG; 7.4 MBq in 0.1 ml of saline solution) was intravenously injected via the tail vein. Then, 60 min after the injection of ^18^F-FDG, PET images were acquired for 30 min using the following parameters: axial field of view 94.7 mm (1 bed), 1–5 acquisition coincidence mode, and coincidence time window of 5 ns. The sinogram of the images was reconstructed using the 3D expectation maximization PET reconstruction algorithm (Mediso Tera-Tomo TM, iteration = 4, subset = 3). All PET images were corrected for emission scatter and attenuation. To the quantitative analysis, regions of interest (ROIs) were drawn into the frontal cortex, cortex, hippocampus, and hypothalamus regions using PMOD 3.8 software (PMOD Technologies LLC., Zurich, Switzerland). Subsequently, the regional uptake was calculated as the mean standardized uptake value (SUV) based on the following equation for the quantitative comparison of ^18^F-FDG uptake.

$$ SUV=\frac{tissue\kern0.17em radioactivity\kern0.17em concentration}{injected\kern0.17em activity/ body\kern0.17em weight} $$

### Transmission electron microscopy (TEM)

Prefrontal cortex samples were cut into 1-mm^3^ cubes and immediately fixed in 2.5% glutaraldehyde (Sigma-Aldrich, St. Louis, MO, USA) in 0.1 M phosphate buffer (pH 7.2). Then, samples were postfixed in 4% osmium tetroxide (Sigma-Aldrich) with 3% potassium ferrocyanide in 0.1 M cacodylate buffer (pH 7.3) for 1 h at 4 °C in the dark and embedded in Epon 812 after dehydration in an ethanol and propylene oxide series. Ultrathin slices (70 nm thick) were obtained using an ultramicrotome (Ultracut UCT, Leica, USA) and collected on 150 mesh copper grids. After staining with 2% uranyl acetate for 10 min and lead citrate for 5 min, the sections were observed under a transmission electron microscopy (Tecnai G2 Spiri TWIN; Thermo Fisher Scientific) at 120 kV.

### Statistical analysis

Data are presented as the mean ± standard error of the mean (SEM). The results were analyzed by one-way analysis of variance followed by the Newman-Keuls post hoc test for multiple comparisons using Prism v.5.0 (GraphPad, La Jolla, CA, USA). Statistical significance thresholds were set at *p < 0.05, **p < 0.01, and ***p < 0.001.

## Results

### EA attenuates cognitive impairments in 5XFAD mice

**Fig. 1:**
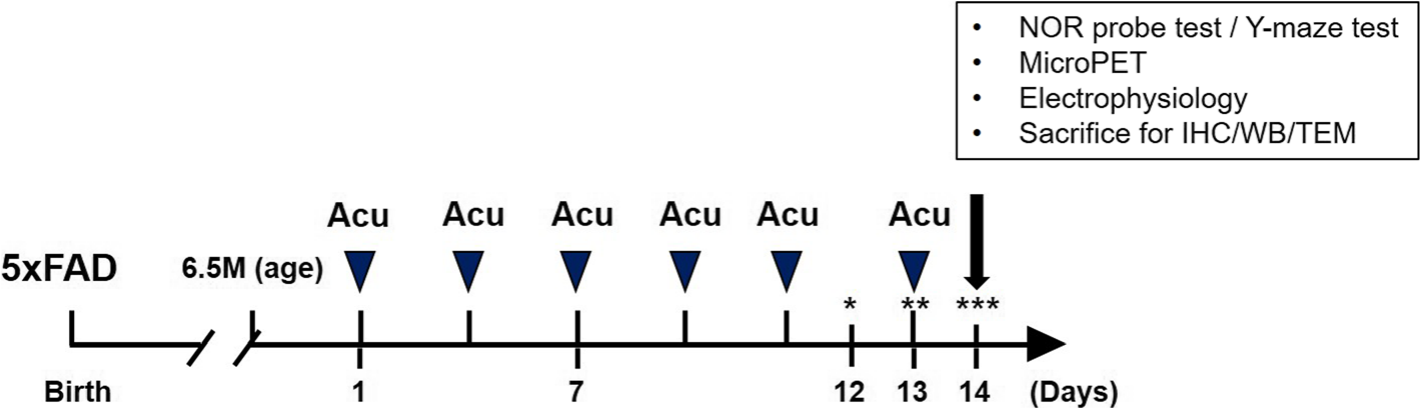
Experimental procedures. NOR novel object recognition test, Acu acupuncture treatment, IHC immunohistochemistry, WB western blot, TEM transmission electron microscopy. An asterisk indicates habituation trial, two asterisks indicate training trial, and three asterisks show probe trial in the NOR test

**Fig. 2:**
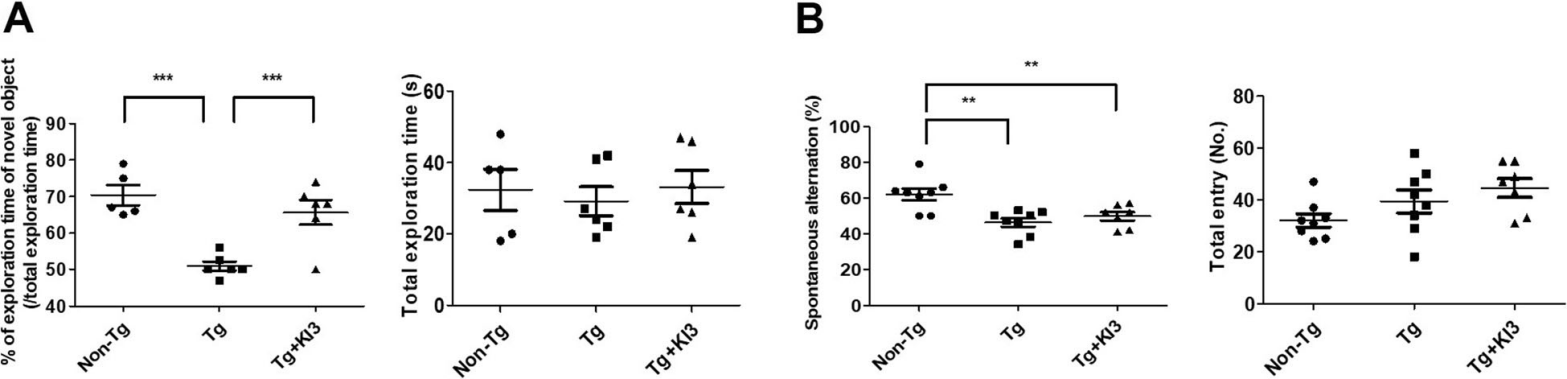
EA treatment alleviates cognitive impairments in 5XFAD mice. EA was applied bilaterally to the KI3 acupoints for 15 min (1 mA, 2 Hz) three times per week for 2 weeks. The NOR probe trial was conducted on the day after the last EA treatment. **a** Data show the results of the NOR test (percentage of exploration time of the novel object and total exploration time; *n* = 5–6/group). **b** Results of the Y-maze test (spontaneous alternations and total entries; *n* = 7–8/group). Data are presented as means ± SEM (**p < 0.01, ***p < 0.001)

EA treatment at KI3 was performed in 6.5-month-old 5XFAD mice three times per week for 2 weeks (Fig. 1). NOR and Y-maze tests were used to evaluate whether EA treatment affects cognitive impairments in 5XFAD mice. We found that the Tg group of untreated 5XFAD mice showed a 1.4-fold reduction in exploration time of novel objects compared to that of non-Tg mice. However, EA treatment increased the mean exploration time of novel objects by 1.3 times compared to that of Tg mice in the NOR test that measures the working memory (Fig. 2a). In the Y-maze test, the total activity was not different among the three groups, non-Tg, Tg, and Tg + KI3. In this test, 5XFAD mice showed a 1.3-fold decrease in the number of spontaneous alternations compared to that of age-matched non-Tg mice indicating an impairment of the hippocampus-dependent spatial memory (Fig. 2b). However, EA treatment did not rescue the observed dysfunction of hippocampus-dependent spatial memory in 5XFAD mice (Fig. 2b). Taken together, these data suggest that EA treatment restored in the 5XFAD model the working memory but not the spatial memory that is dependent on hippocampus functions.

### EA activates specific brain regions in 5XFAD mice

**Fig. 3:**
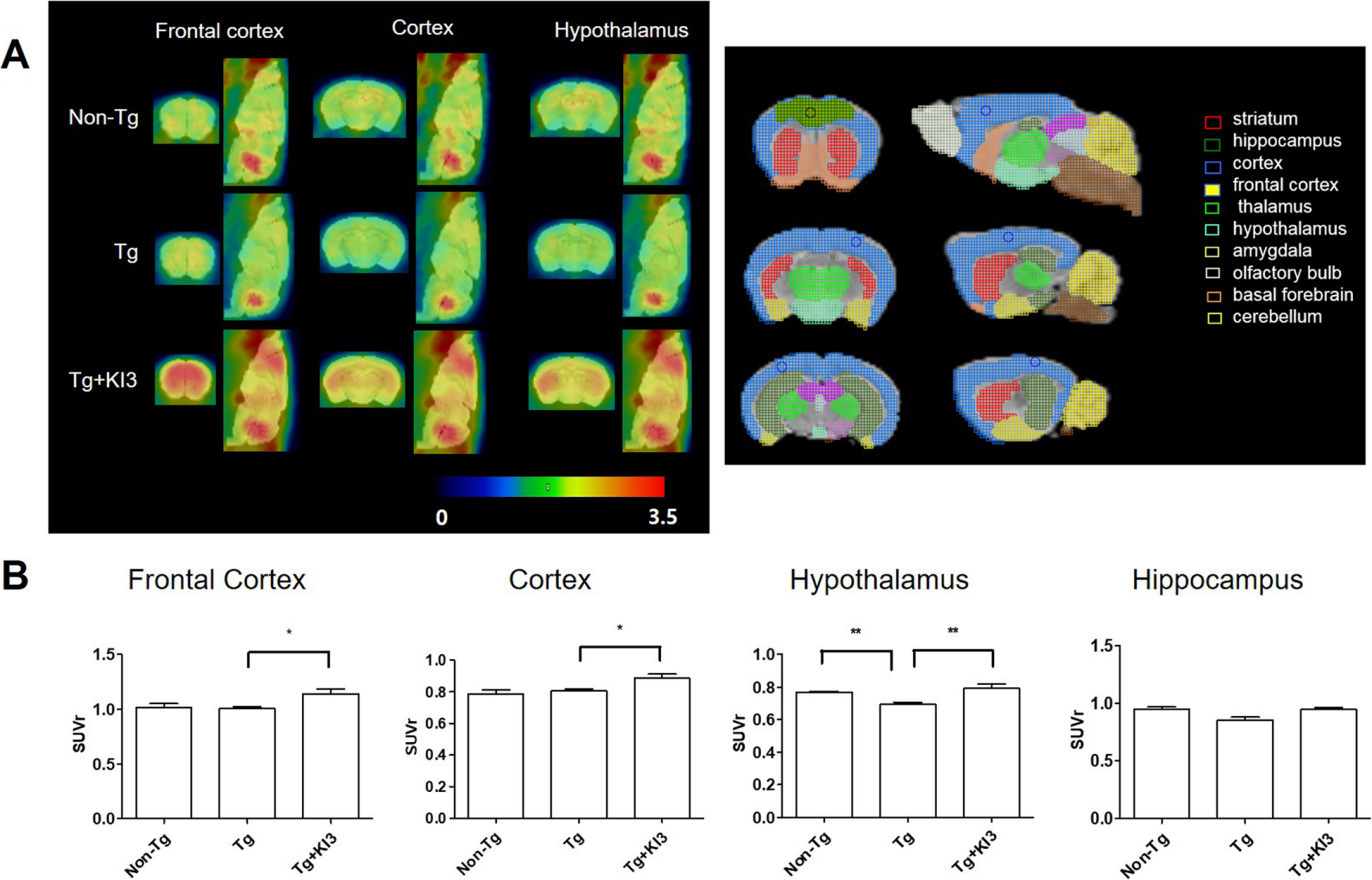
EA treatment increases the metabolic activity of 5XFAD mice as determined by microPET scans. **a** Representative microPET images in specific brain regions (frontal cortex, cortex, and hypothalamus) at 60 min after intravenous injection of ^18^F-FDG on the day after the last EA treatment. The right panel shows the reference for the specific brain region. **b** Quantitative analysis of ROI-based ^18^F-FDG uptake (SUV) in the frontal cortex, cortex, hypothalamus, and hippocampus of each group (*n* = 3–4/group). Data are presented as means ± SEM (*p < 0.05, **p < 0.01)

Some papers reported that acupuncture at KI3 activates the brain to increase cognition in elderly patients with mild cognitive impairment (MCI) [22, 23]. To evaluate whether EA treatment activates the prefrontal cortex region involved in cognitive functions, we investigated glucose metabolism levels using microPET to measure brain functions in 5XFAD mice. As expected, EA stimulation caused a 1.1-fold increase in the mean glucose level of the frontal cortex, which is related to short-term and working memory, compared to that of the Tg group, whereas the levels in the hippocampus did not change (Fig. 3a, b). Furthermore, we observed that the hypothalamus, which is related to energy metabolism, exhibited a 1.1-fold decreased glucose metabolism in Tg mice compared to non-Tg mice, and this effect was fully reversed in EA-treated Tg mice (Fig. 3a, b). These data indicate that EA treatment leads to an activation of the brain metabolism in Tg mice.

### EA attenuates neuroinflammation in 5XFAD mice

**Fig. 4:**
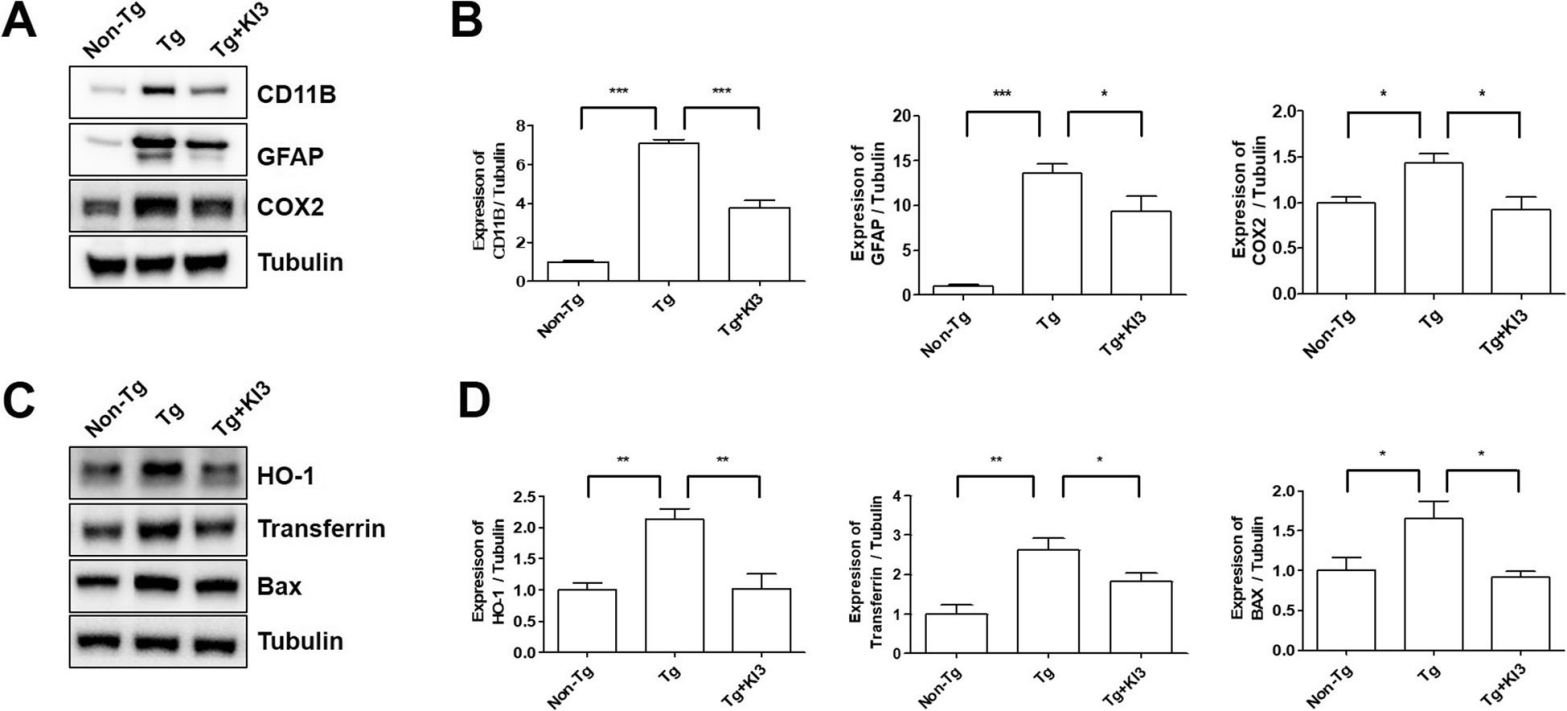
EA treatment attenuates neuroinflammation and decreases the expression levels of oxidation-related proteins in 5XFAD mice. **a** Representative blots for the expression of neuroinflammatory proteins (CD11b, GFAP, and COX2) from non-Tg, Tg, and EA-treated Tg (Tg + KI3) mice. Tubulin was used as the loading control. **b** Quantification of the CD11b, GFAP, and COX2 expression levels (*n* = 3–4/group). **c** Representative immunoreactive bands showing the expression of oxidation-related proteins (HO-1, transferrin, and Bax) in the prefrontal cortex. Tubulin was used as the loading control. **d** Quantification of the HO-1, transferrin, and Bax expression levels (*n* = 3–4/group). Data are presented as means ± SEM (*p < 0.05, **p < 0.01, ***p < 0.001)

Neuroinflammation is a critical factor causing cognitive impairments in 5XFAD mice or AD [24]. To investigate the molecular mechanisms responsible for EA-induced the improvement of cognition, we examined the expression levels of neuroinflammation and oxidative stress-related proteins in the prefrontal cortex of 5XFAD mice. As shown in Fig. 4a, b, the expression levels of CD11b (for microglia), GFAP (for astrocytes), and COX2 showed in the prefrontal cortex of EA-treated mice a significant 1.9-fold, 1.5-fold, and 1.6-fold reduction, respectively, in comparison to the values determined in Tg mice (Additional file 1: Figure S1b and S1d). In addition, EA treatment decreased the levels of the oxidation-related proteins HO-1, transferrin, and Bax 2.1-fold, 1.5-fold, and 1.8-fold, respectively, in the prefrontal cortexes of 5XFAD mice in comparison to those of Tg mice (Fig. 4c, d and Additional file 1: Figure S1a–b). These data suggest that the observed cognitive improvement by EA treatment may be closely related to anti-inflammatory mechanisms in the brain.

### EA increases synaptic plasticity and reduces synaptic ultrastructural degradation in 5XFAD mice

**Fig. 5:**
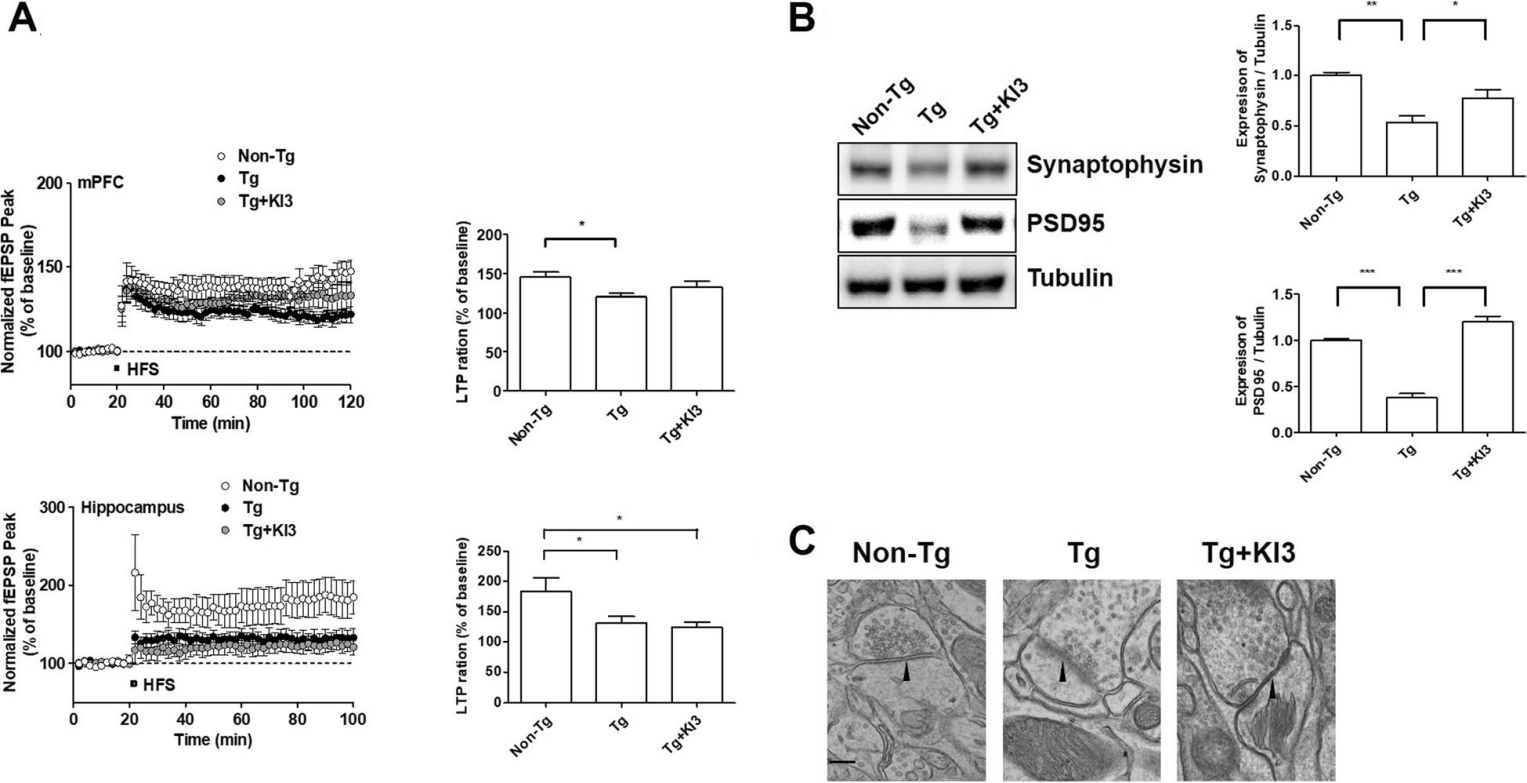
EA increases synaptic plasticity and reduces synaptic ultrastructural degradation in 5XFAD mice. **a** EA treatment increases long-term potentiation (LTP) in the prefrontal cortex and hippocampus of 5XFAD mice. The peaks of field excitatory synaptic potentials (fEPSPs) were normalized using baseline recordings (*n* = 6/group; **a**, left). The graph shows cumulative data displaying the average fEPSP of 90–100 min in the prefrontal cortex and hippocampus after EA treatment (*n* = 6/group; **a**, right). **b** Representative images of immunoblots displaying increased synaptic marker protein levels (synaptophysin and PSD-95) after EA treatment in 5XFAD mice. Tubulin was used as the loading control. Quantitative analysis of the expression levels of synaptophysin and PSD-95 (*n* = 3–4/group). **c** Representative transmission electron microscopy images of synaptic structures (pre- and postsynapse, synaptic space, and synaptic vesicles). Black arrowheads indicate the clearly visible synaptic cleft. Scale bar, 200 μm. Data are presented as means ± SEM (*p < 0.05, **p < 0.01, ***p < 0.001)

Neuroinflammation such as microglia activation leads to a decrease in synaptic plasticity and synapse loss [25]. To investigate the relationship between neuroinflammation and synaptic plasticity after EA treatment, we measured LTP and determined whether synaptic plasticity is influenced by the upregulation of synapse-related proteins in the murine prefrontal cortex. Prefrontal cortex and hippocampus slices of non-Tg mice presented the expected LTP levels (Fig. 5a). The mean LTP level in prefrontal cortex and hippocampus slices from 5XFAD mice showed a significant 1.2-fold and 1.4-fold reduction, respectively, compared to those from non-Tg mice. EA treatment enhanced the LTP level in the prefrontal cortex of 5XFAD mice without reaching statistical significance in comparison to untreated animals, but not in the hippocampus (Fig. 5a). In agreement with these data, we found that stimulation by EA led to a significant 1.4-fold increase in the expression level of synaptophysin in the prefrontal cortex compared to that of untreated Tg mice (Fig. 5b and Additional file 1: Figure S1e). In addition, a significant 3.2-fold increase in PSD-95 protein level was found in EA-treated Tg mice compared to that of untreated Tg mice (Fig. 5b and Additional file 1: Figure S1d). Next, we determined whether EA treatment affects the synapse ultrastructure using transmission electron microscopy in the prefrontal cortex of 5XFAD mice. Synapses of non-Tg mice showed clear synaptic membranes with synaptic vesicles and postsynaptic densities (Fig. 5c). However, 5XFAD mice showed disrupted membranes at pre- and postsynaptic densities, fusion of the synaptic cleft, and loss of synaptic vesicles. By contrast, EA treatment clearly restored the visible synaptic structures compared to those observed in Tg mice (Fig. 5c). These findings indicate that EA upregulated the expression of synaptic proteins and inhibited the degradation of synaptic ultrastructures, thus improving synaptic plasticity.

### EA modulates microglia-mediated Aβ deposition in 5XFAD mice

**Fig. 6:**
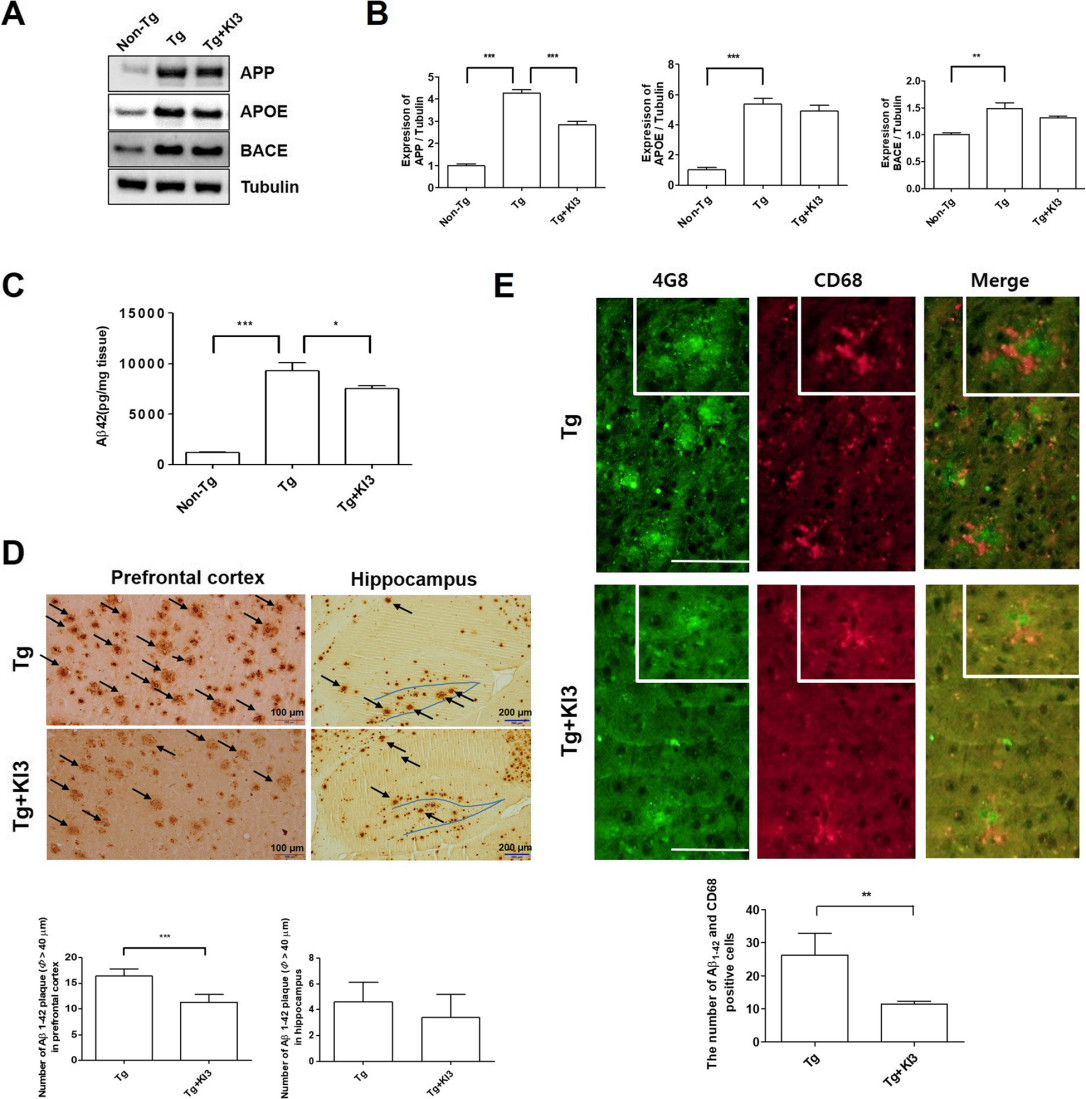
EA treatment modulates microglia-mediated Aβ deposition in 5XFAD mice. **a** Representative western blot images displaying the protein expression of APP, APOE, and BACE in the frontal cortex of mice from each group. Tubulin was used as the loading control. **b** Quantitative analysis of the immune blots normalized to tubulin (*n* = 3–4/group). **c** Insoluble Aβ_1–42_ levels in the murine prefrontal cortex, as determined by ELISAs (*n* = 4–5/group). **d** Representative images showing anti-Aβ (4G8) immunostaining in the prefrontal cortex and hippocampus region of 5XFAD mice. The black arrow indicates Aβ plaque with a diameter > 40 μm (**d**, upper). Quantification of Aβ plaques in the prefrontal cortex and hippocampus (*n* = 5/group; **d**, down). Scale bar, 100 μm and 200 μm. **e** Representative images of immunofluorescence staining using anti-Aβ (4G8) (green) and anti-CD68 (red) in the prefrontal cortex region of 5XFAD mice. The block of photomicrographs display typical CD68-positive cell in Aβ plaques (**e**). Quantitative analysis of the number of CD68^+^ cells/Aβ plaque (*n* = 5/group; **e**, down). Scale bar, 100 μm. Data are presented as means ± SEM (*p < 0.05, **p < 0.01, ***p < 0.001)

To determine whether EA affects Aβ depositions in the prefrontal cortex of 5XFAD mice, we studied the protein expression levels related to Aβ production and the amounts of insoluble Aβ in western blots and ELISAs. As shown in Fig. 6a, the expression levels of proteins related to Aβ_1–42_ production such as APP, APOE, and BACE showed a significant 4.3-, 5.4-, and 1.5-fold increase, respectively, in the prefrontal cortex of Tg mice relative to the endogenous levels of non-Tg mice (Additional file 1: Figure S1a and S1c). However, EA treatment caused a 1.5-fold reduction in the level of APP protein compared to that of untreated Tg mice. In addition, the levels of APOE and BACE were reduced after EA treatment without reaching statistical significance (Fig. 6a, b and Additional file 1: Figure S1c). To determine whether EA treatment affects insoluble Aβ_1–42_ levels and Aβ_1–42_ plaques in the prefrontal cortex, we performed Aβ_1-42_ ELISAs and immunohistochemistry. EA treatment significantly lowered 1.2-fold the insoluble Aβ_1–42_ levels in the prefrontal cortex of Tg mice (Fig. 6c). In agreement with these ELISA results, EA treatment led in the prefrontal cortex to a 1.5-fold reduction in the number of Aβ_1–42_ plaques with a diameter > 40 μm compared to the values determined in untreated Tg mice (Fig. 6d). However, EA treatment did not reduce the number of Aβ_1–42_ plaques in the hippocampus of Tg mice (Fig. 6d). Amyloid depositions are reportedly surrounded by activated microglia [26]. Based on this paper, we investigated the colocalization of Aβ_1–42_ plaques and CD68-positive cells in the prefrontal cortex of 5XFAD mice to determine the relationship between a reduction in Aβ plaque and neuroinflammation after EA treatment. As shown in Fig. 6e, EA treatment significantly reduced 2.3-fold the number of colocalized Aβ_1–42_ and CD68 (microglia marker) in the prefrontal cortex of 5XFAD mice. These data indicate that EA treatment attenuates microglia-mediated Aβ_1–42_ deposition in the prefrontal cortex of Tg mice.

## Discussion

In past decades, the incidence of cognitive impairment has been increasing due to various reasons, and many people focus now on the prevention of cognitive impairments that may lead to dementia. EA as one of the complementary alternative medicine (CAM) techniques showed clinically neuroprotective effects in patients with mild cognitive impairment [27]. In addition, the effectiveness of EA for cognitive improvement in AD and vascular dementia is supported by clinical and animal studies [28, 29]. However, there has been no report yet on the mechanisms linking changes in cognition and neuroinflammation in EA-treated AD animals. In the current study, we revealed for the first time the EA-induced molecular mechanisms responsible for the correlation between cognitive improvement and anti-inflammation in the brains of 5XFAD mice, an animal model of AD.

5XFAD mice reveal hippocampus- or cortex-dependent cognitive impairments in behavioral tests such as the Y-maze test or contextual fear conditioning [30, 31]. The prefrontal cortex is important for recognition memory, facilitating the association between novel and familiar objects [32]. In our study, we confirmed that cognitive deficits in 5XFAD mice were hippocampus- and cortex-dependent. In the NOR test, EA treatment significantly improved the hippocampus-independent working memory in 5XFAD mice. In hippocampus-dependent cognition, EA treatment showed a trend to effectiveness without reaching statistical significance. This indicates that a longer EA treatment may be necessary to reverse this impairment. These data suggest that EA treatment activates rather the prefrontal cortex area than the hippocampus in 5XFAD mice because only a short-term EA treatment for 2 weeks was performed in this study. In agreement with these data, Girard et al. reported that early cognitive deficits in 5XFAD mice are related to the frontal cortex before hippocampus-dependent impairments are detectable [31]. However, it needs to be investigated in further studies whether long-term EA treatment can also recover the hippocampus-dependent long-term memory.

Using microPET, we also observed that EA treatment activated the frontal cortex and the hypothalamus in 5XFAD mice. These results indicate that EA treatment modulates the abnormal hypothalamic metabolism and contributes to the energy metabolism, thus improving cognitive functions in 5XFAD mice. This is supported by Zheng et al. demonstrating that brain metabolism abnormalities are mainly detected in the hypothalamus and that these disturbances of the hypothalamus metabolism are related to the amyloid pathology in AD mice [33]. Recently, Wang et al. reported that adiponectin regulates glucose and lipid metabolism, as well as insulin sensitivity in the hypothalamus, hippocampus, and cerebral cortex, and improves cognitive functions by an increase in synaptic functions and LTP in 5XAFD mice [34]. In addition, they demonstrated that adiponectin treatment modulates apoptotic signaling including activated caspase 3 and nuclear factor kappa-light-chain-enhancer of activated B cells (NF-κB) and glycogen synthase kinase 3 beta (GSK3β) activation. Furthermore, Guo et al. showed that EA treatment increases the level of adiponectin production through adiponectin receptor 1-mediated GSK3β activation [35]. Therefore, we suggest that cortex and hypothalamus activation by EA treatment may increase adiponectin production and glucose metabolism thus leading to improved cognitive functions in 5XFAD mice.

Neuroinflammation, indicated by the presence of activated microglia, is a critical factor contributing to AD pathogenesis. Many markers including pro-inflammatory cytokines released by activated microglia result in degenerative changes of neurons and are associated with amyloid plaques [36]. Several papers demonstrate that EA-induced anti-inflammatory actions improve cognition and suppress neuroinflammatory factors such as TNF-α and IL-1β involved in toll-like receptor (TLR) signaling in animal models of vascular dementia [19, 37, 38]. In addition, Feng et al. show that EA treatment suppresses the expression levels of pro-inflammatory cytokines related to cognitive decline, including IL-1α, IL-6, and TNF-α [13]. Consistent with these publications, our data demonstrate that EA treatment attenuates neuroinflammation-related proteins (CD11b, GFAP, and COX), as well as oxidation-related proteins (HO-1, transferrin, and Bax), in the prefrontal cortex of 5XFAD mice. These data suggest that EA treatment can regulate synaptic plasticity and the synapse structure based on its modulation of neuroinflammation.

Synaptic plasticity regulates structures and functions of synapse [39]. In AD mice, a loss of synapses is observed and leads to learning and memory impairments [40]. Acupuncture improves spatial learning and memory and prevents changes in dendritic structures in the SAMP8 model [41]. Jing et al. show EA-induced improvement of cognition via LTP enhancement in a rat model of memory impairment [42]. In addition, Li et al. demonstrate that synaptic plasticity is regulated by *N*-methyl-d-aspartate receptor and mammalian target of rapamycin signaling pathways [43, 44]. In support of this, the current study reveals that EA treatment improves synaptic plasticity in 5XFAD mice via an increase in synaptic proteins such as synaptophysin and PSD-95, as well as via reduced ultrastructural degradation. Synaptophysin as a marker of presynaptic plasticity plays a critical role in the release of neurotransmitters, whereas PSD-95 is a postsynaptic marker [45]. Therefore, further studies are necessary to determine the signaling pathways involved in the neurotransmitter-dependent improvement of synaptic plasticity and cognition after EA treatment of 5XFAD mice.

In AD patients and the corresponding animal models, Aβ deposition is detected in both the hippocampus and the cerebral cortex, resulting in learning and memory impairments [46, 47]. Neuroinflammation in AD is related to Aβ accumulation in glial cells such as microglia and astrocytes, thus affecting synaptic plasticity [14, 48]. Activated microglia are involved in Aβ production and clearance in the brain of AD mice [49]. Zhong et al. demonstrated in an animal model of AD that the microglial surface receptor triggering receptor expressed on myeloid cells 2 (TREM-2) plays as a critical role in the protection of neurons by enhancing microglial activities against Aβ depositions and their related toxicity [50]. Based on our results, we conclude that EA treatment significantly decreases the amount of insoluble Aβ in the prefrontal cortex of 5XFAD mice. Furthermore, we observed that EA treatment significantly reduced smaller and compact microglia than microglia colocalized with Aβ plaque in control 5XFAD even though the plaques in EA-treated 5XFAD mice are associated with the remaining microglia. Taken together, our data indicate that EA treatment reduces the number of microglia-mediated Aβ plaques.

Vereker et al. showed that the pro-inflammatory cytokine IL-1β reduces LTP by inhibition of glutamate release in aged rats [51]. Moreover, Feng et al. demonstrated that EA treatment improves cognitive impairments by alleviating neuroinflammation via the microglial TLR2/4 signaling pathway in an animal model of post-operative cognitive dysfunction [51]. In the context of these studies, our data suggest that EA treatment promotes microglia-mediated Aβ degradation and alleviates neuroinflammation via the reduction of pro-inflammatory cytokines in 5XFAD mice. In further studies, it remains to be shown whether the EA-induced alleviation of neuroinflammation and amyloid deposition is dependent on either the frequency of EA treatments or other parameters such as the duration of each session.

## Conclusions

This study provides scientific evidence for anti-inflammatory effects in EA-treated 5XFAD mice, although it remains controversial whether anti-inflammation should be the main target to treat AD patients because anti-inflammatory drugs such as prednisone and rofecoxib failed in clinical studies with AD patients [52, 53]. Reduction in neuroinflammation is the therapeutic target of EA in AD, and EA may ameliorate Aβ-related pathologies and activate signaling pathways responsible for the improvement of cognitive functions in AD mice.

## Supplementary information

**Additional file 1: Figure S1.** Images of the full western blots from prefrontal cortex samples of 5XFAD mice. (a) HO-1, APP, Transferrin, and tubulin (*n* = 3–4/group). (b) CD11B, COX2, BAX, and tubulin (*n* = 3–4/group). (c) BACE, APOE and tubulin (*n* = 3–4/group). (d) GFAP, PSD95 and tubulin (*n* = 3–4/group). (e) Synaptophysin and tubulin (*n* = 3–4/group).)

## Data Availability

The datasets used and/or analyzed in the current study are available from the corresponding author on reasonable request.

## Abbreviations

(HO)-1: Heme oxygenase
(TNF)-α: Tumor necrosis factor
5XFAD: Five familial AD
AD: Alzheimer’s disease
APOE: Apolipoprotein E
APP: Amyloid precursor protein
Aβ: Amyloid beta
Bax: B cell lymphoma-2 (Bcl-2)-associated X protein
CD11b: Cluster of differentiation 11b
EA: Electroacupuncture
fEPSP: Field excitatory postsynaptic potential
GFAP: Glial fibrillary acidic protein
HFS: High-frequency stimulation
KI3: Taegye
LTP: Long-term potentiation
PSD-95: Postsynaptic density-95
ROI: Regions of interest
SUV: Standardized uptake value
