# Supplementary figures and images for: Electroacupuncture attenuates cognition impairment via anti-neuroinflammation in an Alzheimer’s disease animal model
Source: J Neuroinflammation. 2019 Dec 13;16:264. doi: 10.1186/s12974-019-1665-3 (PMC6909515; doi:10.1186/s12974-019-1665-3)

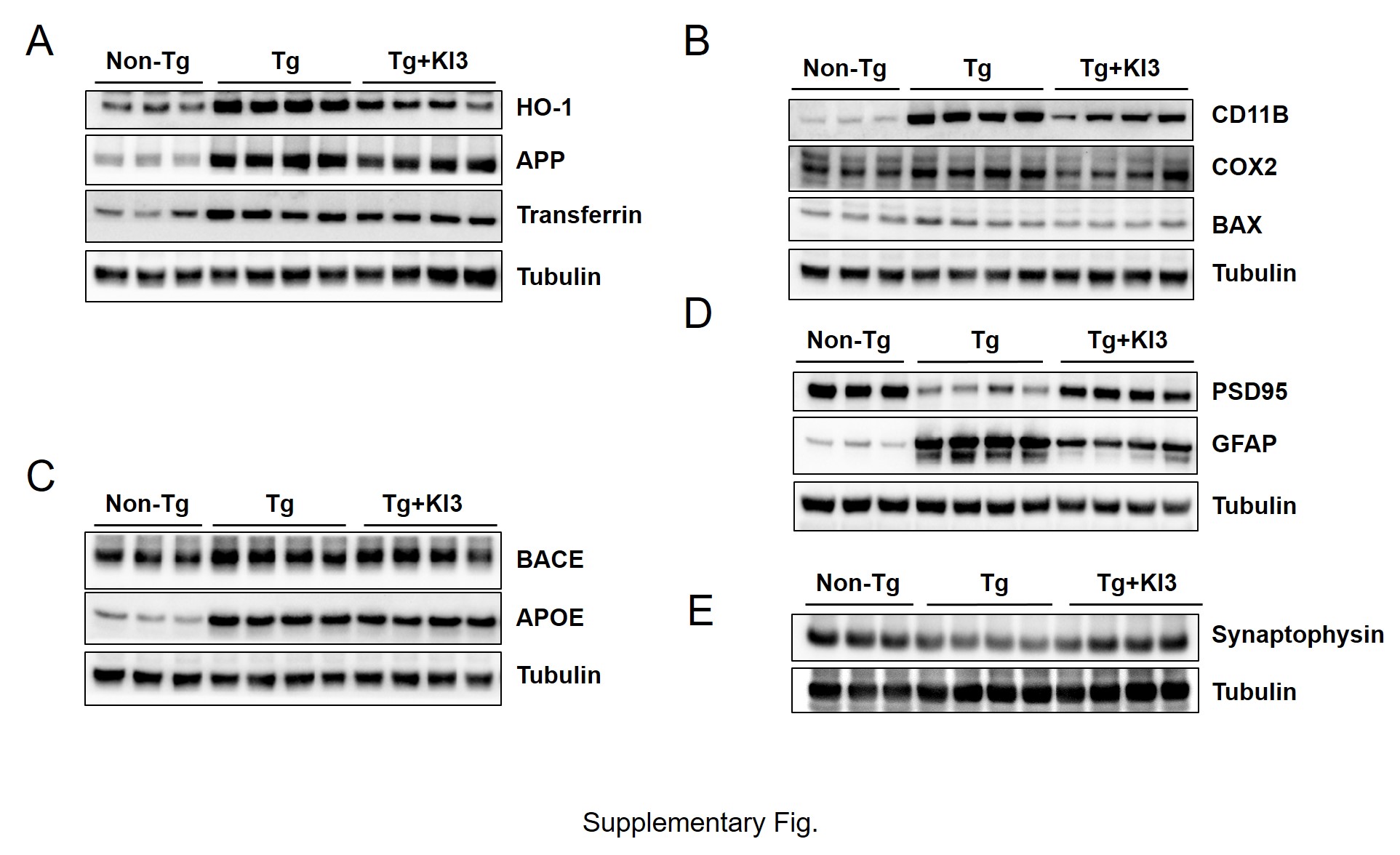

Supplement: Supplementary file 1 — Additional file 1: Figure S1. Images of the full western blots from prefrontal cortex samples of 5XFAD mice. (a) HO-1, APP, Transferrin, and tubulin (n = 3–4/group). (b) CD11B, COX2, BAX, and tubulin (n = 3–4/group). (c) BACE, APOE and tubulin (n = 3–4/group). (d) GFAP, PSD95 and tubulin (n = 3–4/group). (e) Synaptophysin and tubulin (n = 3–4/group).) [file 12974_2019_1665_MOESM1_ESM.jpg]
